# Everybody's Page

**Published:** 1893-02-18

**Authors:** 


### Everybody's Page.

The pauper houses of the future will soon become positively attractive in the eyes of the poor, if matters go on improving as they bid fair to do to-day. The Islington Workhouse has just caused a supply of tobacco to be ordered in for the weekly consumption of its male inmates, whils the Board are discussing also some equivalent form of comfort for the female element of the establishment.

"What's in an eye?" A science paper informs us that in Greek statuary the male eye is represented decidedly more prominent than the female. Nowadays uniformity of surface is not to be observed in either sex, and we doubt if any series of composite photographs would produce any such characteristic difference between the visual organs of the sexes. Phrenologists, we believe, class artistic powers with the prominent eye. From practical observation we are inclined to associate it with shortness of sight.

Tragic as is the present condition of Zante, the "Fair Flower of the Levaut" as it is called, yet its circumstances of to-day are little worse than others which it has already undergone during this century; in 1822 a severe earthquake shook its foundations, demolishing many hundreds of houses, and 20 years later, in 1841, the whole place lay in a mass of ruins. In fact the inhabitants of this island have never felt much security in their home, and have ever been in terror of a repetition of these terrible upheavals of nature; for underneath the fair exterior of olive gardens, vineyards, and fruit trees lurks many a deadly enemy in the form of sulphurous springs, petroleum and tar wells, which are ever ready to make a deadly havoc amongst the islanders..

In Thuringia there is a whole district which is dependent for its support on the manufacture of artificial eyes; husbands, wives, and children all working together at this same means of livelihood. And yet, though these simple German village people turn out their produce by the dozen, no two eyes are ever the same. No artificial eye has its exact fellow either in colour or in size in the whole world. The method of the manufacture is not a very complicated art. There are firstly glass plates, which are blown by gas jets, then moulded by hand into the form of an oval-shaped cup. Then there is the colouring of the eyes, which is effected by the means of tracing with fine needles, the tints being left to the taste of the individual worker, though the scope of their taste is necessarily limited to greys and blues, and browns and blacks, which colours are assorted together before being eventually dispatched to their various destinations.

Many are the changes which our streets have undergone in the varied histories of their paving. Indeed, besides finding sermons in stones, we can find histories, too, if we choose to look. The long life of the cobble-stone paving pointed to healthy nerves and small traffic. Sedan chairs and good nerves expired together, and their death evolved the new life of the more silent thoroughfares of to-day. Latter-day nerves and exaggerated traffic both combined to necessitate some expedient for the intolerable burden of noise. Macadamised roads and wooden pavements conduced to bring this about, and, so far, these changes have satisfied us; but an age of progress is ever an age of unrest, and we are told now by roadway authorities in Germany and in Australia that the great paving medium of the future is made of indiarubber—that no substance is more perfectly adapted for horse and pedestrian traffic. This certainly possesses one great recommendation in our eyes from a sanitary point of view, and that is that indiarubber is a non-conducting microbe medium.

It would be interesting to know what effect, if any, the prohibition of rags from infected districts, such for instance as Hamburg a month or so back, has had upon the manufacture of paper generally. The import of rags is by no means an insignificant one now-a-days in the rapidly-increasing production of paper. Up to 1725 there were only two paper mills in America. Now we are told the yearly import of rags alone has reached the high figure of 121,000,000 pounds, which equals about 65,500 tons of that commodity.

The favoured queen of an Eastern harem has her creature comforts very carefully looked after. An amusing account of one of her meals coming before our notice the other day, we append it for the benefit of our readers. The menu had, as its *pièce de résistance*, a repast consisting of a young lamb roasted whole, stuffed with a turkey, which again was stuffed with a chicken, stuffed with a pigeon, the pigeon on its part being stuffed with a quail, and lastly this quail was stuffed with one of the smallest of Eastern birds called a fig-pecker. It is hard to imagine what was the prevailing flavour of this much amassed dish—certainly suggestive as it is of the last flight of an epicurean's brain.

An extraordinary tale comes from over the seas. An American vessel reports that Pitcairn Island is experiencing an extraordinary drought. It is said that no rain has fallen for two years, and that the island is drying up. The inhabitants, some 130 in number, are subsisting on nothing but fruit, and the only animals they possess are dogs and cats, runs the tale. The island is of volcanic origin. 'Tis a lesson for the socialist to reflect upon, this inequality in natural distribution. How well we could do with a little less and the inhabitants of Pitcairn with a little more. Perhaps a little musketry practice on the part of the natives would cause the cloud above the island to prove more yielding.

A Parisian Opera-Singer v. The Telephone is a somewhat curious legal case lately brought before the French capital. Whether it is the telephone itself who will be the defendant or the telephone's company will require the finer decision of the law to decide. Let the plaintiff define his own case. He says that the engagement he had entered into at the opera was to sing to the audience assembled within its walls, not outside of them; whereas this offender, which we allude to as the defendant, on the opposition side, had, without permission, transmitted the singer's efforts to various communicating centres to which it was attached. This the plaintiff affirms to be injurious to him from a financial point of view, and hence his expostulation; but how far the human voice can be patented has got to be decided.

It has been known from remote times in the history of medicine how diametrically opposite effects can be produced from the same drug through the amount of it prescribed. Indeed, the importance of the size of a dose in changing therapeutic action, is a subject which is still open to much debate; the particular sect who have hitherto taken it up having deduced from the study a somewhat false basis of thought. Amongst those medicines which have so varying a difference in their effect when administered in large or small quantities, we may briefly mention rhubarb, chloride of sodium, calomel, ipecac, sweet spirit of nitre, and antimony, these drugs respectively being the agents of quite opposite results according to their individual prescription. Text books have hitherto had little to say on this subject, and yet it is one of considerable significance, and worthy of more importance than it has yet been treated with.

